# Exploring potential relationships between acoustic indices and ecosystem functions: a test on insect herbivory

**DOI:** 10.1007/s00442-024-05536-9

**Published:** 2024-04-06

**Authors:** Francesco Martini, You-Fang Chen, Christos Mammides, Eben Goodale, Uromi Manage Goodale

**Affiliations:** 1 Botany Department, School of Natural Sciences, Trinity College Dublin, Dublin, Ireland; 2 State Key Laboratory of Vegetation and Environmental Change, Institute of Botany, Chinese Academy of Sciences, Beijing, 100093 China; 3 Nature Conservation Unit, Frederick University, 7, Yianni Frederickou Street, Pallouriotissa, 1036 Nicosia, Cyprus; 4 Department of Health and Environmental Science, Xi’an Jiaotong Liverpool University, Suzhou, China

**Keywords:** Soundscape ecology, Biodiversity monitoring, Ecosystem processes, Forest regeneration, Insect diversity

## Abstract

Biodiversity loss is a global concern. Current technological advances allow the development of novel tools that can monitor biodiversity remotely with minimal disturbance. One example is passive acoustic monitoring (PAM), which involves recording the soundscape of an area using autonomous recording units, and processing these data using acoustic indices, for example, to estimate the diversity of various vocal animal groups. We explored the hypothesis that data obtained through PAM could also be used to study ecosystem functions. Specifically, we investigated the potential relationship between seven commonly used acoustic indices and insect leaf herbivory, measured as total leaf damage and as the damage from three major insect feeding guilds. Herbivory was quantified on seedlings in 13 plots in four subtropical forests in south China, and acoustic data, representing insect acoustic complexity, were obtained by recording the evening soundscapes in those same locations. Herbivory levels correlated positively with the acoustic entropy index, commonly reported as one of the best-performing indices, whose high values indicate higher acoustic complexity, likely due to greater insect diversity. Relationships for specific feeding guilds were moderately stronger for chewers, indicating that the acoustic indices capture some insect groups more than others (e.g., chewers include soniferous taxa such as crickets, whereas miners are mostly silent). Our findings suggest that the use of PAM to monitor ecosystem functions deserves to be explored further, as this is a research field with unexplored potential. Well-designed targeted studies could help us better understand how to best use novel technologies to monitor ecosystem functions.

**Supplementary Information:**

The online version contains supplementary material available at 10.1007/s00442-024-05536-9.

## Introduction

Biodiversity loss is one of the biggest global crises. Species extinction rates continue to increase to unprecedented levels, leading to what has been described as the Sixth Mass Extinction (Ceballos et al. 2015; Cowie et al. 2022). Importantly, biodiversity is not only important per se, but it is closely linked to the function and maintenance of resistant and resilient ecosystems (Hooper et al. 2005; Pennekamp et al. 2018). It is imperative to investigate further the relationships between biodiversity and ecosystem functions, and to explore the potential applications of novel technologies in doing so.

In recent years, there have been significant improvements in technological solutions used to remotely monitor biodiversity, for example, through the use of passive acoustic monitoring (PAM) systems (Alcocer et al. 2022; Sugai et al. 2019). Such tools allow researchers to collect soundscape data by positioning acoustic sensors in the areas of interest, which can then be analyzed to estimate the number of vocal species, identify focal species, or derive indices of acoustic complexity, which may correlate with species diversity (Alcocer et al. 2022). Despite unresolved limitations and recommended caution in the use and interpretation of their exact relationships to biodiversity, several of these indices generally correlate well with biodiversity monitored in the field. In terrestrial systems, the indices have been mostly used for bats and birds (Alcocer et al. 2022; Sugai et al. 2019). More recent efforts suggest that the recordings can also capture other soniferous animals, such as insects (Aide et al. 2017; van Klink et al. 2022), although these examples are less common (Sugai et al. 2019). Building on increasing access to such data, researchers are starting to explore new approaches to take advantage of these novel technologies to answer ‘old’ ecological questions (Ross et al. 2023). For example, we may be able to monitor ecosystem functions such as pollination or wood use by birds (e.g., for communication by woodpeckers) using data collected through PAM (Desjonquères et al. 2020; Folliot et al. 2022).

Ecosystem processes and functions depend on biodiversity (Balvanera et al. 2006). In forests, herbivore consumption of organic matter contributes to the transformation and movement of nutrients in the environment (Metcalfe et al. 2014; Ramirez et al. 2021). In tropical and subtropical forests, insects make up for most of these nutrients’ flow (Metcalfe et al. 2014). Variation in herbivory levels on plants’ leaves has been traditionally linked to the diversity and composition of neighboring plant individuals, leaf traits, and the indirect effects of environmental variables, such as light and soil nutrients, which affect plants’ investments in palatable and defensive compounds (Loney et al. 2006; Schuldt et al. 2012). Although less understood, some evidence suggests that the diversity of herbivorous insects correlate to the damage observed on leaves, and to the number of types of damage (Bito et al. 2011; Bustos-Segura et al. 2017; De Carvalho Guimarães et al. 2014; Eichhorn et al. 2007). Greater diversity of insects and related feeding guilds likely increases the number of species capable of consuming different plant species and leaf structures, potentially leading to greater overall damage to leaves (Neves et al. 2010).

We tested the hypothesis that information extracted from acoustic recordings correlates to ecosystem function, using data on insect leaf herbivory as our response variable. Specifically, we were interested in assessing whether commonly used indices of acoustic complexity derived from sound recordings, assumed to represent insect diversity, would positively correlate to herbivore damage on leaves measured using traditional field methods. In addition, we assessed whether the correlation would be more prominent for insect groups comprising mostly soniferous species as opposed to mostly silent groups, which we identified by examining the various forms of damage (such as chew marks, mining, and sucking) on the leaves of seedlings.

## Methods

### Study area

We used data collected in a network of thirteen 1-ha forest monitoring plots (Table 1) distributed in four National Nature Reserves in the Guangxi Zhuang Autonomous Region in south China: (1) Cenwanglaoshan (24°21’N, 106°27’E); (2) Dayaoshan (23°52’N, 110°01’E); (3) Mulun (25°07'N, 107°54’E); and (4) Huaping (25°36’ N, 109°50’E). The permanent plots, setup and monitored by Guangxi University, are situated in areas of intact relatively undisturbed forest. While logging or similar major disturbance activities are not permitted in these forests, some plots are relatively close (though more than 90 m) to moderately trafficked mountain roads, and people may also pass by on footpaths, or more rarely pass inside the plots.

**Table 1:** Summary information of the studied forest reserves and sampling periods for herbivory and soundscape recordings

Forest reserve	Forest type	Plots	Elevation (m asl)	Temperature (°C)^a^	Precipitation (mm)^a^	Herbivory measurements	Soundscape recordings
Cenwanglaoshan	Deciduous broad-leaf mixed forest	5	1365–1842	14	1857	August 2018	April–May 2016
Dayaoshan	Broadleaf evergreen forest	4	530–1320	17	1824	August 2018	May–June 2017
Huaping	Deciduous broad-leaf mixed forest	2	809–870	19	1500	August 2018	May–July 2019
Mulun	Mixed evergreen deciduous broadleaf karst forest	2	368–569	13	2100	August 2018	April–June 2017

Information obtained from Wen et al. (2004) and Du et al. (2017)

^a^Mean annual value

### Data collection

For the purposes of this project, we combined parts of three data sets that have been previously published from these study plots. Two studies provided the herbivory data on total leaf damage (Martini et al. 2021) and on the occurrence of insect feeding guilds on the same seedlings’ leaves (Martini & Goodale 2020), while the third provided data on acoustic indices (Chen et al. 2021). Below, we provide a brief overview of the methodology used for the measurements of herbivory and acoustic data, but we refer to the original studies for more detailed descriptions.

### Herbivory

Leaf herbivory was measured on the seedling community in each 1-ha plot. Seedlings were tagged and identified in eight census stations per 1-ha plot. Each census station consisted of three 1 m^2^ quadrats, where all woody seedlings (excluding lianas) ≤ 50 cm tall were tagged, measured, and identified to species or morpho-species. In August of 2018, all leaves were visually assessed (*N* = 8072) in all tagged seedlings (*N* = 1377) and quantified the percentage of each leaf that was eaten or damaged by herbivorous insects. Each leaf was assigned to a percentage class (0%, 1–5%, 6–25%, 26–50%, 51–75%, and > 75%), and later, the intermediate percentage value of each interval was used (0%, 2.5%, 15%, 37.5%, 62.5%, and 87.5%) to estimate the percentage of leaf damage for each seedling by taking the mean (Martini et al. 2021). Furthermore, the occurrence of insect-feeding guilds that had attacked each leaf was assessed by the morphology of the leaf damage (see Figure S2 in Martini et al., 2020 for an example of damage types). We considered six guilds, but only used three for analysis: chewer, miner, and suckers. Rollers, gallers and skeletonizers were measured but not analyzed here, because they were rare (Martini et al., 2020).

### Soundscape recordings

The data set from Chen et al. (2021) includes acoustic data of soniferous forest birds and insects collected during the morning and the evening in the plot network during breeding seasons from mid-April to mid-August in 2016 to 2019. Because we were interested in the relationship between insect diversity and herbivory, we only used the data from the evening recordings that were collected after sunset and mainly captured the peak activity of insects (Chen et al. 2021). Nine autonomous acoustic recorders Song Meter model SM3 (Wildlife Acoustics, Maynard, MA, USA) were used, equipped with omnidirectional microphones. The recorders were attached to tree trunks with a rope at ca. 1.5 m height approximately at the center of each 1-ha plot. They recorded sounds 1 h after sunset for 10 min (Shonfield & Bayne 2017), for at least 30 consecutive days at each site. All recordings were saved in.wav format at a sample rate of 24,000 Hz (16 bits). Each plot was only sampled in 1 year.

For each 10-min recording, seven acoustics indices used commonly in the literature were calculated (a description of each index is available in Table 2): Acoustic Complexity Index (ACI, Pieretti et al. 2011), Acoustic Diversity Index (ADI), Acoustic Evenness Index (AE, Villanueva-Rivera et al. 2011), Bioacoustic Index (BIO, Boelman et al. 2007), Normalized Difference Soundscape Index (NDSI, Kasten et al. 2012), Acoustic Entropy Index (H, Sueur et al. 2008a, b), and Acoustic Richness Index (AR, Depraetere et al. 2012). The *soundecology* (for the first five indices) and *seewave* (for H and AR) packages were used in the R statistical environment (Sueur et al. 2008a, b; Villanueva-Rivera & Pijanowski 2022). The availability of these indices in well-known R packages has made them the most applied indices (Alcocer et al. 2022). Because each index reflects some different properties of the soundscape, we retained all indices in the following analysis, as is commonly done and recommended by other studies (Bradfer-Lawrence et al. 2019; Mammides et al. 2017).

**Table 2:** Description of the seven acoustic indices used in this study

Reference	Index	Properties
Pieretti et al. (2011)	Acoustic Complexity Index (ACI)	It measures the variability in amplitude between adjacent frequency bins
Villanueva-Rivera et al. (2011)	Acoustic Diversity Index (ADI)	It uses the Shannon–Weiner diversity index to calculate acoustic diversity based on the frequency bands of the spectrograms. It ranges from 0 to the log of the number of frequency bands in the spectrogram
Villanueva-Rivera et al. (2011)	Acoustic Evenness (AE)	It uses the Gini coefficient based on the same frequency bands used by ADI. Bounded between 0 and 1, with higher values indicating lower acoustic evenness
Depraetere et al. (2012)	Acoustic Richness (AR)	It is based on the temporal component of the acoustic entropy index and the median of the amplitude envelope
Boelman et al. (2007)	Bioacoustic Index (BIO)	It calculates acoustic complexity by measuring the variation in signal intensity relative to the frequency band with the lowest amplitude, with greater differences between bands giving a higher value
Sueur et al. (2008a, b)	Acoustic Entropy (H)	It uses the Shannon–Weiner index to calculate spatial and temporal entropy, based on the frequency bands selected to divide the recordings. The spatial and temporal components are then multiplied to obtain the final value. Bounded between 0 and 1, with 0 indicating pure tones and 1 indicating high-energy
Kasten et al. (2012)	Normalized difference soundscape index (NDSI)	It is the ratio between anthrophony (1–2 kHz, assumed to be mostly made by human disturbance), and biophony (2–11 kHz, assumed to be mostly made by living species). Bound between − 1 and 1, with higher values signifying lower disturbance

### Statistical analysis

We summarized herbivory and the acoustic indices at the plot level (*N* = 13). For herbivory, we used mean herbivore damage (%) from all measured seedlings in each 1-ha plot. Likewise, for each acoustic index, we calculated the mean value for each plot across the 30 days (Chen et al. 2021). To test the potential relationship between the acoustic indices and herbivory, we used linear mixed models with the R package *glmmTMB* (Brooks et al. 2017). We ran one model for each acoustic index, thus seven models in total. In each model, herbivore damage was the response variable, and each acoustic index was the explanatory variable. We used forest reserve as a random effect in each model, which allowed us to account for the effect of general elevation, as well as other reserve-specific characteristics. In preliminary analyses, we found that elevation did not have an additive effect when added as a covariate in the models; therefore, we left elevation out of the subsequent analysis. Herbivory was ln(x + 1) transformed to meet the assumptions of normality and homogeneity of variance required for linear models, which we assessed using the R package *DHARMa* (Hartig 2022). We calculated the marginal and conditional R^2^ for each model using the ‘r.squaredGLMM’ function from the *MuMIn* R package (Bartoń, 2022).

To model the potential relationship between the acoustic indices and the herbivory caused by each of the three selected insect guilds, recorded as the number of leaves attacked by each guild, we used generalized linear mixed models, also using *glmmTMB*, with a beta-binomial distribution. For each guild, as a response variable, we used a two-column matrix with the number of attacked leaves (success) and the number of undamaged leaves (failure) per plot using the function ‘cbind’. As done for herbivore damage, we ran a model for each acoustic index as the explanatory variable, and we used forest reserve as a random effect. Thus, we ran seven models for each guild, and 21 models in total. We used R version 4.3.0 for all analyses (R Core Team 2023).

## Results

Leaf herbivory showed, on average, 14% damage across all individual seedlings and varied between 9.9% and 21.1% at the plot level, with significant variation between plots (Figure S2). The acoustic indices were, for the most part, weakly or moderately correlated among each other (10 relationships |*r*|< 0.5; 10 relationships |*r*|≥ 0.5, < 0.7; one relationship, between AE and ADI |*r*|= − 0.96; Figure S1). The different indices showed different patterns of variation across plots, with the acoustic complexity index (ACI) and the acoustic richness index (AR) having no significant differences, while the other indices having wider and significant differences (Figure S3).

**Fig. 1:**
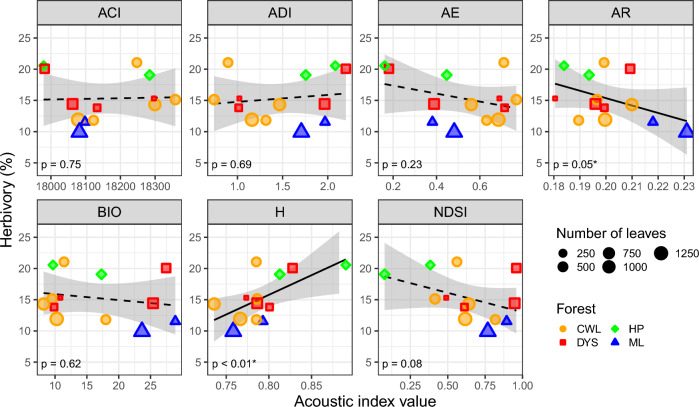
Relationship between herbivory and each individual acoustic index. Stronger relationships (*p* < 0.05) are shown with solid lines, while dashed lines indicate weak to no relationships (*p* > 0.05). The shaded area around the regression line represents the 95% confidence intervals. The *p* values correspond to the result of the linear mixed models. Plots from the same forest are represented with the same color and shape. The size of each point is proportional to the number of leaves measured for herbivory. Forest sites are Cenwanglaoshan (CWL), Dayaoshan (DYS), Huaping (HP), and Mulun (ML)

We found a positive relationship between the acoustic entropy (H) index and herbivory (*p* < 0.01; *R*^2^_m_ = 0.39, *R*^2^_c_ = 0.39), as well as evidence of a negative relationship with AR (*p* = 0.045; *R*^2^_m_ = 0.25, *R*^2^_c_ = 0.25; Fig. 1; Table S1). H is bound between 0 and 1, with higher values indicating increasing acoustic complexity in sound recordings and, thus, greater diversity. AR is also bound between 0 and 1, with values closer to 1 representing higher acoustic complexity and potentially species diversity (Depraetere et al. 2012), and here was negatively related to herbivory. There was also a marginally significant negative relationship between NDSI and herbivore damage (*p* = 0.08; *R*^2^_m_ = 0.20, *R*^2^_c_ = 0.20; Fig. 1; Table S1). NDSI is the ratio between low-frequency sounds (1–2 kHz, assumed to be mostly made by human disturbance), and higher-frequency sounds (2–11 kHz, assumed to be mostly made by living species) and is bound between -1 and 1, with higher values signifying lower disturbance.

**Fig. 2:**
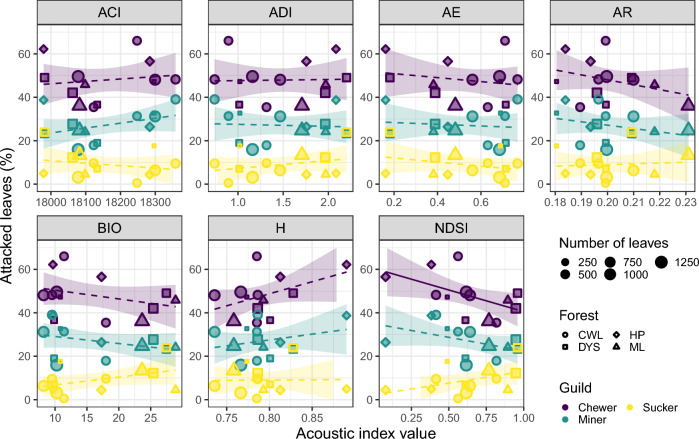
Relationship between the three most common insect feeding guilds and each acoustic index. Stronger relationships (*p* < 0.05) are shown with solid lines, while dashed lines indicate weak to no relationships (*p* > 0.05). The shaded area around the regression line represents the 95% confidence intervals. The color indicates the insect guild type, while the shape of the points indicates the forest site. The size of each point is proportional to the number of leaves measured for herbivory. Forest sites are Cenwanglaoshan (CWL), Dayaoshan (DYS), Huaping (HP), and Mulun (ML)

When analyzing the data by insect feeding guild, our results highlight a negative relationship between the proportion of leaves attacked by chewers and NDSI (*p* = 0.02). Other models showed no significant relationship (Fig. 2, Table S2), although H was marginally positively significant for chewers (*p* = 0.06). Nonetheless, there were some differences in the direction and slope of the relationship among the three studied guilds. Specifically, chewers and miners were more similar, while suckers had opposite directions when tested with some indices, such as NDSI or BIO (Fig. 2).

## Discussion

We tested the hypothesis that insect diversity, inferred from the analysis of PAM recordings and the derived indices of acoustic complexity, correlates with ecosystem functions, specifically insect herbivory. We found significant relationships with total herbivore damage, as well as with individual feeding guilds. These findings support the idea that novel technologies, such as PAM, could potentially be used beyond their conventional use of biodiversity monitoring, towards the understanding and monitoring of ecosystem processes.

Herbivore damage was positively related to the acoustic entropy index (H) and negatively to the acoustic richness index (AR) (Fig. 1). H has been reported in previous studies as the index showing stronger relationships with variables of interest, for example, vegetation characteristics and diversity of birds (Chen et al. 2021; Fuller et al. 2015; Mammides et al. 2017). In general, it is considered one of the indices showing the best performance in measuring biodiversity (Alcocer et al. 2022). Moreover, this finding agrees with previous studies showing that increasing insect diversity is mirrored by increased herbivore damage (Bustos-Segura et al. 2017; Neves et al. 2010), suggesting the potential to connect PAM data to ecosystem functions. Surprisingly, AR was negatively related to herbivory. Negative relationships between insect richness measured in the field and leaf damage have occasionally been reported (Bito et al. 2011). However, AR has been described as a poorly reliable index (Alcocer et al. 2022), including in the study that originally collected the acoustic data we used here (Chen et al. 2021). Furthermore, relationships with AR might not have a lot of biological meaning, because AR did not vary significantly across plots (Figure S3). Therefore, we are cautious about the ability of this index to accurately capture ecosystem processes. Of the other indices, only NDSI showed a marginally significant relationship. NDSI is another index, along with H and ACI, that has been found to be relatively efficient at estimating biodiversity in a recent meta-analysis (Alcocer et al. 2022). However, the direction of the relationships was mostly positive in the meta-analysis just mentioned, while it was negative here. It is also useful to note, however, that these indices are known to show inconsistent relationships with biodiversity among studies, suggesting their performance is area specific (Eldridge et al. 2018; Mammides et al. 2017). The other indices (ADI, AE, BIO) were all reported as weakly connected to biodiversity (Alcocer et al. 2022). Our results partially agree with the previous literature, with most of the indices that are thought to best reflect actual biological diversity also showing stronger relationships with herbivory. At the same time, a possible explanation for the somewhat inconsistent relationships might be that the study is located in a highly biodiverse region, whereas acoustic indices perform better at capturing biodiversity at higher latitudes (Pan et al. 2024). Conducting similar studies to what we have presented here in temperate systems could help clarify which indices perform better.

We expected to find clear differences in the direction of the relationships between acoustic indices and herbivory between feeding guilds. Most evening sounds were produced by crickets (identified by manual listening of the recordings, Table 2 in Chen et al. 2021), which are mostly herbivores, although they can feed on other organic material (Ingrisch & Rentz 2009). Crickets are chewers, and thus, we would expect a stronger correlation between the indices and chewing damage. For this guild, we detected a negative relationship with NDSI, another index considered a good indicator of biodiversity (Alcocer et al. 2022), but H was marginally significant and positive (Fig. 2). These results confirm that acoustic entropy seems to be a reliable index. In addition, our findings indicate that acoustic indices are expectedly limited in capturing damage from insect guilds composed mostly of silent species. For example, leaf-mining insects belong mostly to the orders Lepidoptera, Coleoptera, and Hymenoptera (Hespenheide 1991), which were not detectable on the soundscape recordings (Chen et al. 2021).

Our study presents several limitations that need to be considered. First, the acoustic data and herbivory data were not collected concurrently, resulting in a temporal mismatch. However, herbivore damage did not show major variation across years in one forest, where multiple years of data were available (Figure S4). Acoustic indices generally showed less within-plot variation over different years and seasons (April–mid June versus mid-June through August) than between-plot variation, using data from one reserve not included in this study in which there were two consecutive years of data and 4 months of data in the second year; this indicates that the differences between plots were more influential on the indices than temporal variables (Chen et al. 2021). Second, leaf herbivory is caused by many different insects. Most insect orders do not produce sounds (Greenfield 2016), hence cannot be recorded through PAM. This means that part of the measured damage is produced by consumers that cannot be captured by the recorders. Third, the acoustic recorders were placed at the center of each 1-ha plot, while herbivory was measured in the surroundings (distance of approximately 50–70 m). Therefore, the measurement of soundscape recordings and herbivory are not fully spatially aligned. Yet, because we conducted the analyses at the plot level, this is likely a minor issue.

To conclude, we have provided some preliminary findings that support the hypothesis that novel technologies used to monitor biodiversity, such as PAM, could be applied to improve our understanding of the biodiversity–ecosystem function relationships (Desjonquères et al. 2020). As previously mentioned, our study is preliminary in nature and comes with several caveats, including the use of observational data and a relatively limited sample size. We did not expect to provide definitive findings, but rather we hoped that our study would inspire researchers to explore the potential of acoustic data further to relate to ecosystem function. We recommend some improvements. First, it is necessary to design studies with the objective of specifically investigating the relationship between diversity indices, biodiversity, and ecosystem function, to overcome some of our limitations. Second, the ability to include measurements that could provide valuable information, such as, in our example, insect abundance, would provide additional value. Measurements of herbivory, such as those used here, are relatively simple to carry out and do not require additional equipment. Study areas with similar settings to those used here, like forest permanent monitoring plots, could be ideal candidates to develop well-designed experiments. Future improvements in the capacity of the indices of acoustic complexity to capture biodiversity more accurately will most likely be reflected in stronger ability to capture ecosystem functions (Folliot et al. 2022), such as the herbivory tested here. Overall, we believe that this is a field of research that has the potential to develop and increase our understanding of ecosystems and the interactions between their biotic components.

### Supplementary Information

Below is the link to the electronic supplementary material.

Supplementary file1 (DOCX 726 KB)

## Data Availability

The original data are available at the references indicated in the methods. The aggregated data used for the analyses of this study are available in Figshare: 10.6084/m9.figshare.23626410.

## Code availability

The R code is available from the corresponding author upon a reasonable request.
