# Supplementary material for: Exploring potential relationships between acoustic indices and ecosystem functions: a test on insect herbivory
Source: Oecologia. 2024 Apr 6;204(4):875–83. doi: 10.1007/s00442-024-05536-9 (PMC11062954; doi:10.1007/s00442-024-05536-9)
Supplement: Supplementary file 1 — Supplementary file1 (DOCX 726 KB) [file 442_2024_5536_MOESM1_ESM.docx]

**SUPPLEMENTARY MATERIALS**

**Exploring potential relationships between acoustic indices and ecosystem functions: A test on insect herbivory**

Francesco Martini^1,*^, You-Fang Chen^2^, Christos Mammides^3^, Eben Goodale^4^, Uromi Manage Goodale^4^

^1^ Botany Department, School of Natural Sciences, Trinity College Dublin, Dublin, Ireland

^2^ State Key Laboratory of Vegetation and Environmental Change, Institute of Botany, Chinese Academy of Sciences, Beijing 100093, China

^3^ Nature Conservation Unit, Frederick University, 7, Yianni Frederickou Street, Pallouriotissa, 1036 Nicosia, Cyprus

^4^ Department of Health and Environmental Science, Xi'an Jiaotong-Liverpool University, China

^*^ Correspondence to Francesco Martini: franmart12@hotmail.it

**Table of Contents**

[**Table S1** 2](#_Toc159492066)

[**Table S2** 3](#_Toc159492067)

[**Figure S1** 4](#_Toc159492068)

[**Figure S2** 5](#_Toc159492069)

[**Figure S3** 6](#_Toc159492070)

[**Figure S4** 7](#_Toc159492071)

**Table S1** Values of the regression coefficients, standard errors, p-values, and marginal and conditional R^2^ values for models with herbivore damage as the response variable. The statistically significant p-values (p-value < 0.05) are shown in bold.

| Acoustic index | Estimate | SE | p-value | Marginal R^2^ | Conditional R^2^ |
| --- | --- | --- | --- | --- | --- |
| ACI | 0.0001 | 0.0004 | 0.751 | 0.01 | 0.37 |
| ADI | 0.0511 | 0.1278 | 0.689 | 0.01 | 0.38 |
| AE | -0.3578 | 0.2974 | 0.229 | 0.10 | 0.44 |
| **AR** | -8.05 | 4.02 | **0.045** | 0.25 | 0.25 |
| BIO | -0.0056 | 0.011 | 0.621 | 0.04 | 0.19 |
| **H** | 3.7253 | 1.3418 | **0.006** | 0.39 | 0.39 |
| NDSI | -0.3914 | 0.226 | 0.083 | 0.20 | 0.20 |

**Table S2** Values of the regression coefficients, standard errors, p-values, and marginal and conditional R^2^ values for models with the number of attacked leaves from each insect feeding guild as the response variable. The statistically significant p-values (p-value < 0.05) are shown in bold.

| Guild | Acoustic index | Estimate | SE | p-value | Marginal R^2^ | Conditional R^2^ |
| --- | --- | --- | --- | --- | --- | --- |
| Chewer | ACI | 0.0004 | 0.0009 | 0.68 | 0 | 0 |
|  | ADI | 0.02 | 0.24166 | 0.93 | 0 | 0 |
|  | AE | -0.4819 | 0.6252 | 0.44 | 0.02 | 0.04 |
|  | AR | -12.338 | 8.054 | 0.13 | 0.04 | 0.04 |
|  | BIO | -0.0233 | 0.0143 | 0.10 | 0.04 | 0.04 |
|  | H | 5.338 | 2.883 | 0.06 | 0.05 | 0.05 |
|  | **NDSI** | -0.9656 | 0.4043 | **0.02** | 0.08 | 0.08 |
| Miner | ACI | 0.0012 | 0.0009 | 0.20 | 0 | 0 |
|  | ADI | 0.0706 | 0.2504 | 0.78 | 0 | 0 |
|  | AE | -0.4756 | 0.5879 | 0.42 | 0.01 | 0.01 |
|  | AR | -12.836 | 8.672 | 0.14 | 0.02 | 0.02 |
|  | BIO | -0.0168 | 0.0156 | 0.28 | 0.01 | 0.01 |
|  | H | 3.45 | 3.178 | 0.28 | 0.01 | 0.01 |
|  | NDSI | -0.7024 | 0.4372 | 0.11 | 0.02 | 0.02 |
| Sucker | ACI | -0.0009 | 0.0013 | 0.50 | 0.01 | 0.07 |
|  | ADI | 0.2415 | 0.3348 | 0.47 | 0.01 | 0.08 |
|  | AE | -0.6983 | 0.7953 | 0.38 | 0.01 | 0.08 |
|  | AR | 8.737 | 14.009 | 0.53 | 0.01 | 0.08 |
|  | BIO | 0.012 | 0.0224 | 0.59 | 0 | 0.07 |
|  | H | -0.4973 | 5.1894 | 0.92 | 0 | 0.07 |
|  | NDSI | 0.7492 | 0.7368 | 0.31 | 0.02 | 0.06 |

**
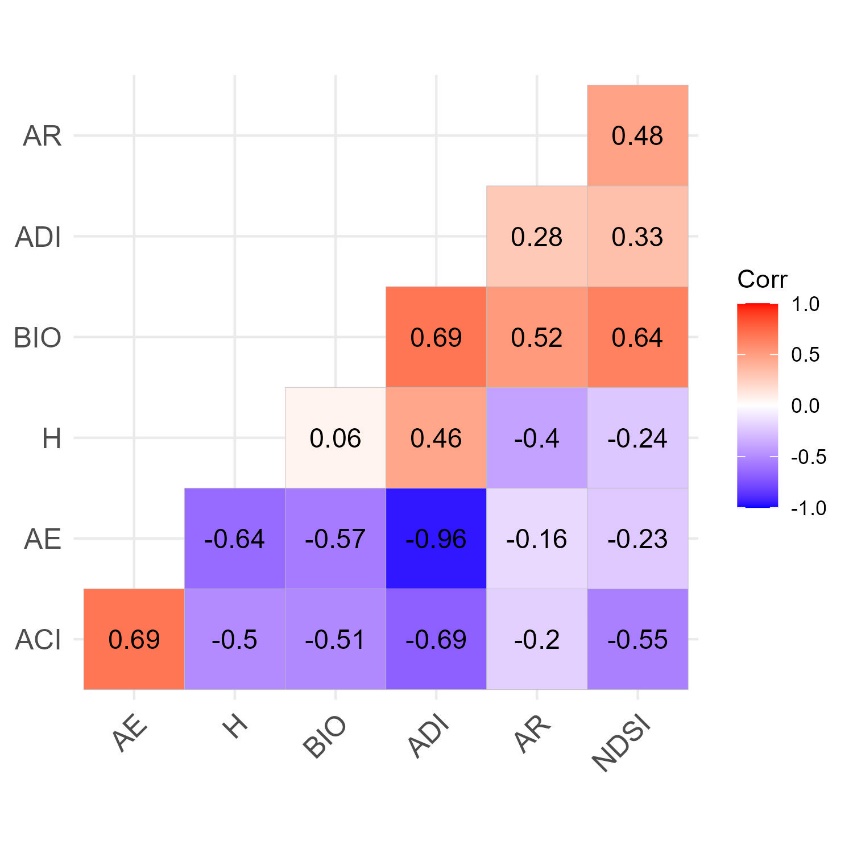
**

**Figure S1** Pairwise Pearson’s correlation values between the seven acoustic indices used in this study. The data used to create the plot are the forest plot values of each index, hence there are 13 data points.

**
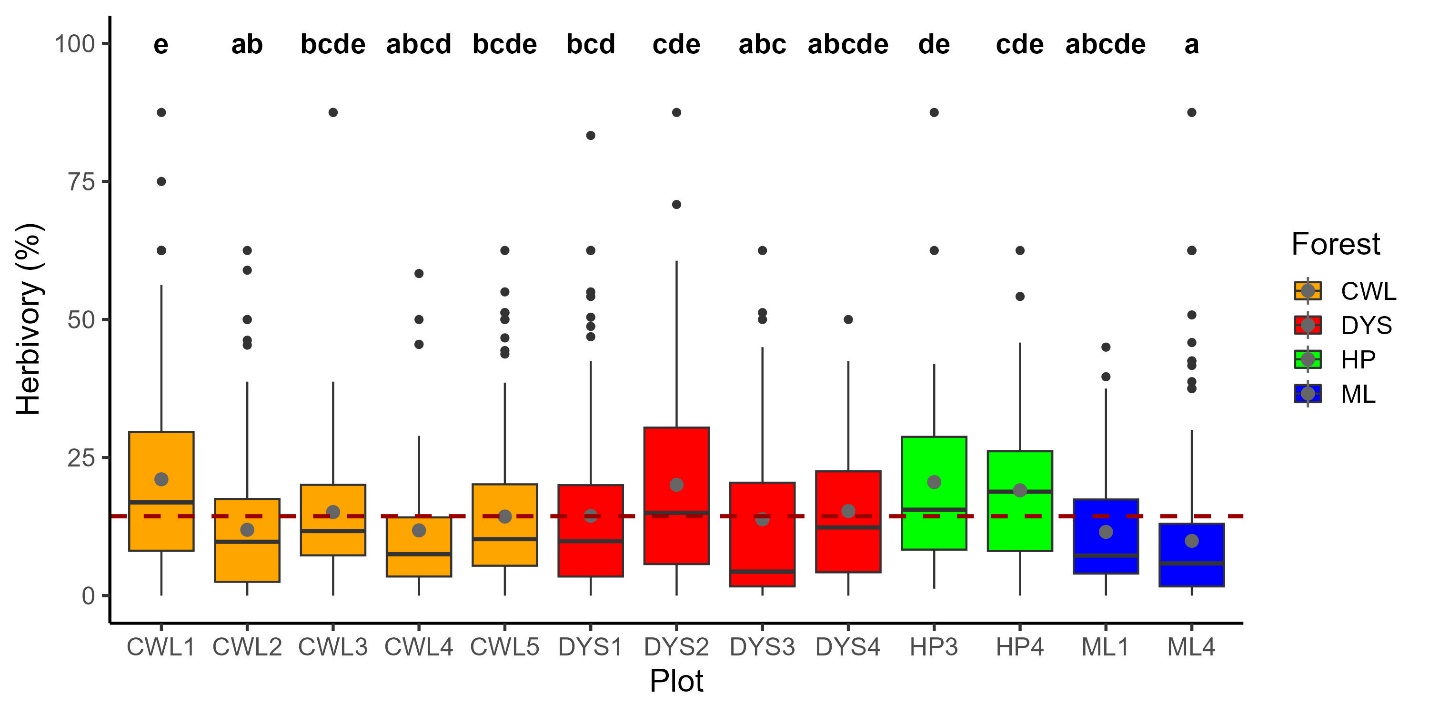
**

**Figure S2** Within and between plot variation in leaf herbivory in the 13 plots used in this study. The grey dots show the mean value for each plot. The dark red dashed line shows the mean leaf herbivory for all plots. The data used are the mean percentage of herbivory for the individual seedlings (n = 1377). Forest sites are Cenwanglaoshan (CWL), Dayaoshan (DYS), Huaping (HP), and Mulun (ML). The letters on top of each boxplot indicated whether the plots are significantly different at p = 0.05. When two plots do not share any letter, they can be considered to have different herbivory (p < 0.05). The results are from a linear mixed model with ‘plot’ as the fixed effect and ‘forest’ as the random effect run with the glmmTMB R package, and the letters were obtained with the ‘emmeans’ and ‘cld’ functions of the *emmeans* (Lenth, 2023) and *multcomp* (Hothorn et al., 2008) R packages, respectively.

**
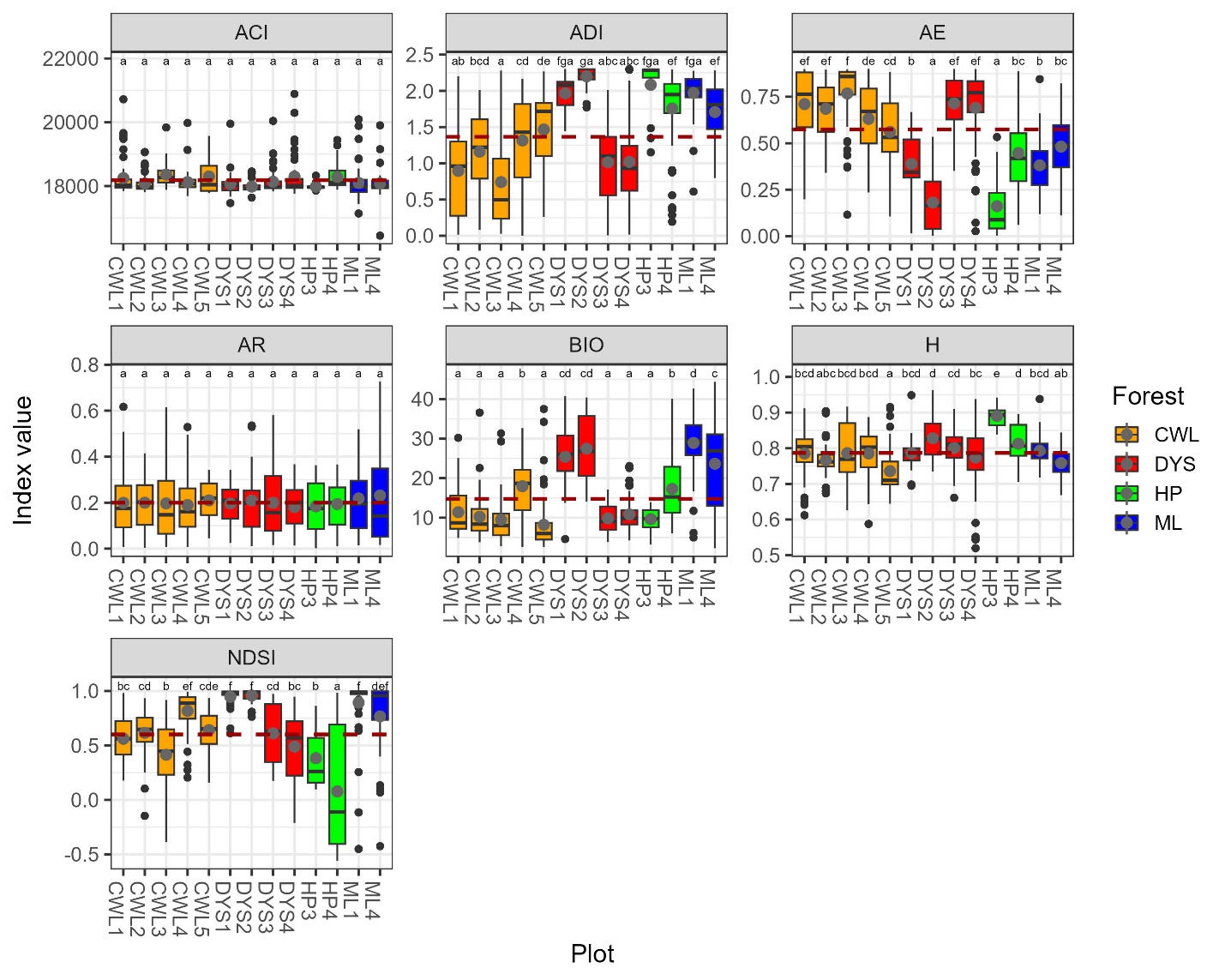
**

**Figure S3** Within and between plot variation for the seven acoustic indices in the 13 plots used in this study. The grey dots show the mean value for each forest plot. The dark red dashed line shows the mean

value of each acoustic index across the 13 plots. The data used are the daily values per forest plot. Forest sites are Cenwanglaoshan (CWL), Dayaoshan (DYS), Huaping (HP), and Mulun (ML). The letters on top of each boxplot indicated whether the plots are significantly different at p = 0.05. When two plots do not

share any letter, they can be considered to have different value of the acoustic index in that panel (p < 0.05). The results are from a linear mixed model with ‘plot’ as the fixed effect and ‘forest’ as the random

effect run with the glmmTMB R package, and letters were obtained with the ‘emmeans’ and ‘cld’ functions of the *emmeans* (Lenth, 2023) and *multcomp* (Hothorn et al., 2008) R packages, respectively.

**
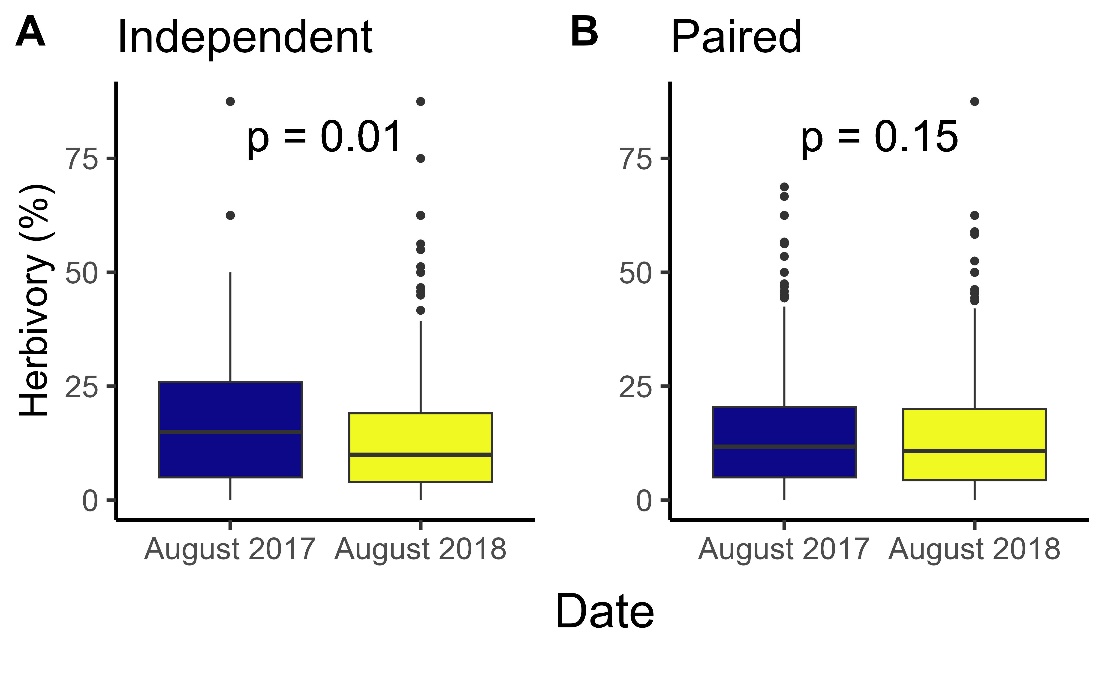
**

**Figure S4** Annual variation in total herbivore damage in the forest site (i.e., Cenwanglaoshan) where two summer measurements were available. **A**) Independent t-test between leaf damage measured in August 2017 and August 2018 on individual seedlings only found at one point in time (N = 669), **B**) Paired t-test between leaf damage measured in August 2017 and August 2018 on individual seedlings found at both points in time (N = 868). Herbivore damage was ln(x+1) transformed.
